# Successful control of heavily pretreated metastatic gastric cancer with the mTOR inhibitor everolimus (RAD001) in a patient with PIK3CA mutation and pS6 overexpression

**DOI:** 10.1186/s12885-015-1139-7

**Published:** 2015-03-13

**Authors:** Ji Hyun Park, Min-Hee Ryu, Young Soo Park, Sook Ryun Park, Young-Soon Na, Baek-Yeol Rhoo, Yoon-Koo Kang

**Affiliations:** 1 Departments of Oncology, Asan Medical Center, University of Ulsan College of Medicine, 88 Olympic-ro 43-gil, Songpa-gu, Seoul, 138-736 South Korea; 2 Department of Pathology, Asan Medical Center, University of Ulsan College of Medicine, 88 Olympic-ro 43-gil, Songpa-gu, Seoul, 138-736 South Korea; 3 Asan Institute for Life Science, Asan Medical Center, University of Ulsan College of Medicine, 88 Olympic-ro 43-gil, Songpa-gu, Seoul, 138-736 South Korea

## Abstract

**Background:**

Everolimus (RAD001) is an orally administered mTOR inhibitor that is well known for its antitumor efficacy and that has been approved for the treatment of several solid tumors, including renal cell carcinoma. In gastric cancer (GC), despite previous preclinical and phase I/II studies suggesting the promising efficacy of everolimus in previously treated AGC, more recent trials revealed that only certain subsets of patients might benefit from treatment with everolimus.

**Case presentation:**

A 26-year-old man with metastatic gastric cancer with multiple liver lesions was treated with everolimus after failure of 1st-line and 2nd-line chemotherapy. A durable partial response was achieved for over 2 years. After progression from initial everolimus treatment, sequential cytotoxic chemotherapies were tried but failed rapidly. Everolimus was re-tried as salvage chemotherapy (re-treatment), and the patient achieved stable disease for 1 year until his death. Subsequent mutational analysis and immunohistochemical (IHC) staining with the tumor tissues just before re-treatment with everolimus revealed a PIK3CA hotspot mutation and pS6 overexpression in the primary tumor. After two cycles of everolimus re-treatment, the overexpression of pS6 became nearly absent in follow-up IHC staining.

**Conclusions:**

Everolimus monotherapy was satisfactory in a patient with refractory metastatic GC harboring PIK3CA and pS6 aberrations. These molecular alterations might be potential biomarkers that can predict the treatment response of everolimus, particularly in the terms of durable disease control. This case suggests and emphasizes that close evaluation of biomarkers in tumor tissue may be essential for identifying highly favorable groups among various subpopulations with AGC.

## Background

The phosphatidylinositol 3-kinase (PI3K)/protein kinase B (PKB or Akt) and mammalian target of rapamycin (mTOR) signaling pathway plays an essential role in cell growth, protein translation, autophagy, and metabolism, which is mainly activated by various growth factor receptors (i.e., HER2 (human epidermal growth factor receptor-2)) or by phosphatase and tensin homolog (PTEN) mutation. Activation of the PI3K/Akt/mTOR signaling pathway is well established to be related to tumorigenesis and cancer progression in many types of tumors, which could further contribute to acquired resistance to various anti-neoplastic agents. In human gastric cancer (GC), PI3K/Akt and mTOR are known to be activated in approximately 30% and 60% of patients, respectively [1,2]. Therefore, in the era of molecular-targeted agents, inhibition of the mTOR pathway represents a novel therapeutic strategy in the treatment of GC.

mTOR is a key down-stream protein kinase of the PI3K/Akt signaling pathway, and everolimus (RAD001) is a novel macrolide derivative of rapamycin that inhibits mTOR, thereby preventing phosphorylation of its downstream molecules. In addition to its promising antitumor efficacy in the treatment of renal cell carcinoma and other several cancer types, the clinical benefit and safety profiles of everolimus in previously treated GC have been explored in several preclinical and phase I/II studies [3-6]. In a more recent phase III trial, however, everolimus monotherapy failed to significantly improve overall survival (OS) in patients with refractory AGC [7]. These intriguing findings suggest that treatment with everolimus may be beneficial only in a subset of GC patients who have been previously treated. In this context, the identification of biomarkers for everolimus seems to be clinically crucial for the selection of patients most likely to benefit from the treatment and ultimately to optimize the efficacy of everolimus.

Herein, we present a case of successful treatment with everolimus in a patient with previously heavily treated AGC, who was found to harbor concurrent dysregulation in PIK3CA and pS6.

## Case presentation

**Figure 1:**
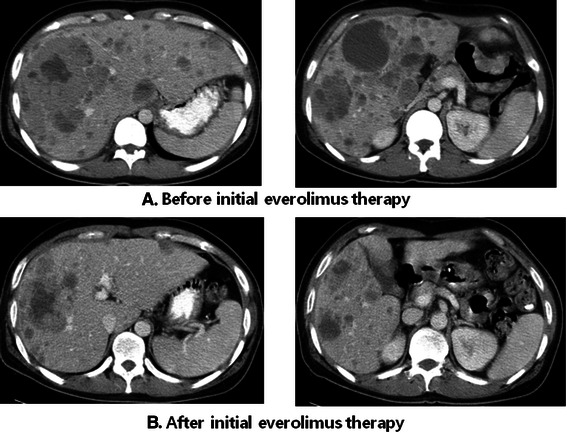
**The patient received initial treatment of everolimus 10 mg/d as the third-line chemotherapy.** CT scans were taken every 2 months during the initial treatment period. CT scans reveal the diffuse liver metastases before treatment with everolimus **(A)**. After 1 year and 2 months of everolimus 10 mg/d, partial response was achieved **(B)**.

A 26-year-old Korean male was diagnosed with stage IV poorly differentiated gastric adenocarcinoma of the lower body with multiple liver metastases in August 2009. The initial diagnosis was made at an outside hospital using abdominopelvic computed-tomography (CT) and endoscopic biopsy of stomach. The pathologic specimen at the time of diagnosis was not procured. The patient was initially treated with systemic chemotherapies, including three cycles of S-1 and subsequently four cycles of FOLFOX chemotherapy, resulting in the progression of liver metastasis. After second-line chemotherapy, the patient was transferred to our institute (Asan medical center, Seoul, Korea) for further management in September 2008. Baseline tumor tissue was obtained via endoscopic forceps biopsy before treatment. As third-line chemotherapy, the patient was enrolled in an open-labeled, phase II trial evaluating the efficacy of everolimus 10 mg/day, and he achieved a partial response by the Response Evaluation Criteria in Solid Tumors (RECIST) as the best response after 1 year and 2 months (Figure 1A and B). The tumor remained stable with a partial response over 2 years in this initial treatment of everolimus. However, after 29 months of initial everolimus treatment, abdominopelvic CT revealed the progression of liver metastasis. An episodic pneumonia due to streptococcus occurred right after disease evaluation, and the patient underwent antibiotic treatment for a month. Subsequently, the patient underwent salvage chemotherapy with FOLFIRI but failed after 3 cycles with disease progression. Sequential three cycles of oral paclitaxel and six cycles of docetaxel chemotherapy were applied to the patient for 4 months to maintain stable disease. During the period of docetaxel treatment, however, the patient suffered from general weakness accompanied by poor compliance and eventually failed the treatment. In such circumstances, we decided to resume the everolimus monotherapy as salvage chemotherapy.

**Figure 2:**
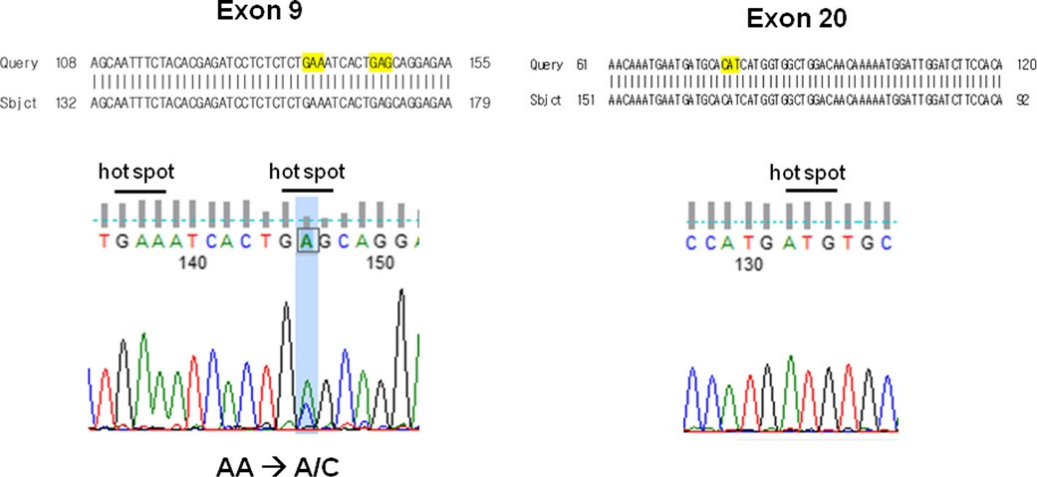
**Mutation analysis of the PIK3CA gene was performed on gastroscopic biopsy specimen obtained before everolimus re-treatment, revealing one of the classical activating PIK3CA mutations at hotspot E545K in exon 9.**

**Figure 3:**
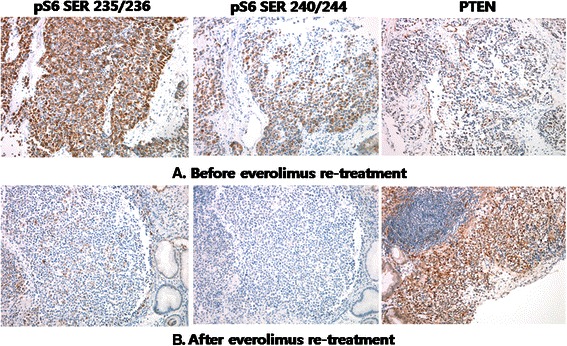
**Immunohistochemical (IHC) staining for pS6 (serine 235/236 and serine 240/244) and PTEN was performed just before and during the everolimus re-treatment.** All photographs were taken at 200 X magnification. Before everolimus re-treatment, the tumor cells exhibited strong positivity for pS6 in more than 50% of the tumor, whereas PTEN was weakly positive in tumor cells compared with the internal controls of endothelial cells **(A)**. After 2 months of everolimus re-treatment, the tumor cells in the follow-up biopsy were completely negative for pS6. However, the intensity of PTEN was notably increased in all tumor cells **(B)**.

**Figure 4:**
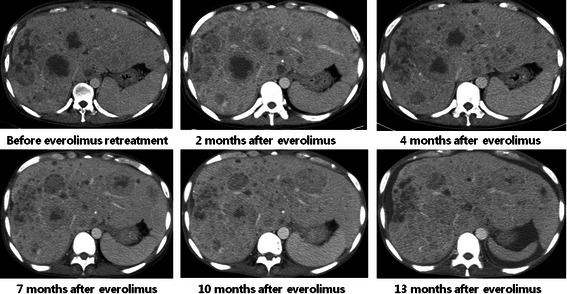
**These serial CT scans demonstrate the efficacy of re-treatment with everolimus 10 mg/d.** The liver metastases remained stable during the period of re-treatment with everolimus for over 2 years.

Before re-treatment with everolimus, according to results from our previous retrospective data [8], we investigated the mutation of the PIK3CA gene in the tumor tissue obtained just before re-treatment of everolimus. DNA was amplified using oligonucleotide primers specific for mutational hotspots in exons 9 and 20 of human PIK3CA. The mutational analysis revealed one of the classical activating PIK3CA hotspot mutations: E545K in exon 9 (Figure 2). In addition, we performed immunohistochemical (IHC) staining for pS6 at serine 235/236 (pS6^Ser235/236^, 1:150 dilution, rabbit monoclonal, Cell Signaling Techmology) and at serine 240/244 (pS6^Ser240/244^, 1:200 dilution, rabbit polyclonal, Cell Signaling Technology) and PTEN (1:50 dilution, rabbit monoclonal, Cell Signaling Technology) with tumor tissues obtained just before and 2 months after re-treatment with everolimus. The tumor cells were strongly positive for pS6 in more than 50% of the tumor, and PTEN was weakly positive in tumor cells relative to the internal controls of endothelial cells (Figure 3A). From January 2012 to February 2013, during the re-treatment with 10 mg everolimus daily, we evaluated the tumor every 2 months by APCT and observed stationary state of liver metastasis. After two months of everolimus re-treatment, the patient underwent follow-up endoscopic biopsy and IHC staining for pS6 and PTEN. Strikingly, the tumor cells in the follow-up biopsy were completely negative for pS6, whereas the intensity of PTEN was notably increased in all tumor cells (Figure 3B). As a result of re-treatment with everolimus, this patient was able to maintain stable disease for more than 1 year but unfortunately died in March 2013 (Figure 4).

## Conclusions

mTOR acts by directly phosphorylating two primary downstream targets of mTORC1, activating ribosomal protein S6 kinase 1 (S6K1) and inhibiting translational repressor 4E-binding protein 1 (4EBP1). S6K1 phosphorylates the S6 protein of the 40s ribosomal subunit at several sites, including Ser 235/236 and Ser 240/244, leading to the initiation of protein synthesis [9]. Everolimus inhibits the ability of mTOR to phosphorylate S6K1 and 4EBP1, thereby inducing G0/G1 arrest and inhibition of cell-cycle propagation in cancer cells [8-10]. Although it is well known that tumors with mutations in the mTOR pathways are uniquely susceptible to mTOR inhibitor therapy, the anti-proliferative efficacy of everolimus depends on the type of cell lines, which exhibit different sensitivities to everolimus [9-11].

Although the first phase II trial on patients with AGC revealed the robust efficacy of disease control rate (DCR) for everolimus [5], the results of the latest phase III GRANITE-1 study were somewhat disappointing. However, this study at the time suggested that everolimus might exhibit activity in a subset of heavily pretreated AGC patients, which urged the development of predictive biomarkers to select the subset of patients with favorable sensitivity for the drug [7]. Therefore, great efforts in last decade focused on exploring the biomarkers for everolimus [8,10,12,13], which identified various potential biomarkers in vitro and in vivo. In several previous studies, PIK3CA/PTEN genomic aberrations were suggested as strong predictors of everolimus sensitivity. The documented mutations of PIK3CA were mostly missense mutations and clustered in two lesions consisting of three hotspots (E542K, E545K, and H1047) [2,10,13-15]. In several preclinical data, loss of staining on IHC was observed in aberrations of PTEN, and the level of expression markedly increased after treatment with everolimus in GC cell lines [14-17]. It is generally accepted assumption with several preclinical data that clinically effective everolimus administration should provide a degree and duration of S6K1 inhibition in peripheral blood and tumor tissues. In these investigations, elevated baseline activity of S6K was related to increased sensitivity toward everolimus [10]. According to our previous phase II study on biomarkers for everolimus, elevated expression of baseline pS6 was significantly associated with higher DCR and progression-free survival (PFS). More interestingly, a relative decrease in pS6 expression by at least 1 IHC score was also an independent predictor of superior PFS in that it might be a relevant index reflecting the extent of mTOR inhibition [8]. Increased levels of pAkt and 4E-BP1 expression were observed to be potential predictive markers in more recent literature [13,18].

Here, we present an intriguing case of heavily pretreated AGC with liver metastases that was successfully controlled over a year with re-treatment with everolimus. While it was reported that rechallenge with mTOR inhibitors can be effective in metastatic renal cell carcinoma patients who progressed on previous mTOR inhibitor therapy [19], this case is the first report of a durable control of metastatic GC with everolimus re-treatment in a patient who was previously treated with the drug. One salient aspect of this case was that the primary tumor was found to harbor one hotspot mutation in PIK3CA and expressed high levels of pS6 (serine 235/236 and 240/244) as well as weakly positive PTEN on IHC staining before everolimus re-treatment. In addition, the baseline pS6 overexpression decreased precipitously after everolimus exposure, whereas the intensity of PTEN was notably increased in follow-up specimens. Considering previous studies on biomarkers, PIK3CA mutation, baseline pS6 overexpression, and loss of PTEN may all have contributed to the successful control of refractory AGC in this patient. Although little was investigated on the significance of reduction of pS6 expression reflecting the decreased activity of S6K, it might be also related to increased sensitivity to everolimus. Hence, these molecular changes might be considered significant predictors for sensitivity or resistance to everolimus. Another encouraging but somewhat confounding aspect was the satisfactory outcome of everolimus monotherapy not only on initial trial but also on salvage treatment with respect to disease control. Everolimus exhibited a DCR of 56% and an objective response of 0% in phase II trials, which suggested that the major role of everolimus in AGC is control of the disease rather than shrinkage of tumor volume [5]. Consistent with these results, our findings revealed reproducible and successful DCR on retreatment with everolimus in a patient harboring potential molecular biomarkers, even the patient experienced disease progression after initial everolimus therapy. The major reason we re-treated the patient with everolimus was the previously achieved satisfactory partial response over 2 years on initial trial. Considering the tumor heterogeneity of GC, we may cautiously speculate that the most sensitive subclones to everolimus would contribute most in achieving durable response during initial treatment. In the context, there would remain relatively sensitive subclones serving a key role in maintaining the stable disease on everolimus re-treatment, These subclones might exhibit higher sensitivity to everolimus than remnant resistant subclones, but not higher than the most sensitive subclones previously responded in initial treatment. Hence, it seems reasonable that the best response of everolimus re-treatment reached only stable disease which was inferior to that of initial therapy [20-23]. Although the mechanism of the resistance to mTOR inhibitor is unclear, disease progression after initial everolimus treatment may be explained by evolving progression mainly due to proliferation of the remnant resistant subclones. Activation of alternative signaling pathway as MAPK pathway in compensation of prolonged mTOR inhibition might possibly take part in emerging resistance to everolimus [24].

Taken together, the prediction of sensitivity to everolimus using tumor tissues might be the most critical aspect on strategy of everolimus treatment, and this should be based upon the reliable biomarkers. Considering that AGC typically proceeds to refractory disease and that prognosis remains disappointing, this suggestion seems to be quite noteworthy in that findings herein may provide a milestone for optimizing the treatment strategies with everolimus.

In summary, this case demonstrated the efficacy of salvage everolimus monotherapy in the disease control of heavily pretreated metastatic GC in a patient with potential biomarkers. More importantly, our results suggest that close evaluation of biomarkers in tumor tissue may be helpful for selecting highly favorable groups among various subpopulations with AGC. Mutational aberrations in PIK3CA and PTEN and highly expressed baseline pS6 (serine 235/236 and 240/244) seem to be significant and reliable biomarkers for predicting the treatment response of everolimus. Thus, in refractory metastatic GC, patients with these biomarkers, even initially treated with everolimus, might be considered potential candidates of salvage chemotherapy with the drug, although further investigation is warranted in a larger population cohort.

## Consent

Written informed consent was obtained from the patient’s wife for publication of this case report and any accompanying images. A copy of the written consent is available for review by the Editor of this journal.
